# HCV Drug Resistance Challenges in Japan: The Role of Pre-Existing Variants and Emerging Resistant Strains in Direct Acting Antiviral Therapy

**DOI:** 10.3390/v7102876

**Published:** 2015-10-13

**Authors:** Kazuaki Chayama, C. Nelson Hayes

**Affiliations:** 1 Department of Gastroenterology and Metabolism, Applied Life Sciences, Institute of Biomedical and Health Sciences, Hiroshima University, 1-2-3 Kasumi, Minami-ku, Hiroshima 734-8551, Japan; nelsonhayes@hiroshima-u.ac.jp; 2 Laboratory for Digestive Diseases, Center for Genomic Medicine, Institute of Physical and Chemical Research (RIKEN), Hiroshima 734-8551, Japan; 3 Liver Research Project Center, Hiroshima University, Hiroshima 734-8551, Japan

**Keywords:** hepatitis C, direct-acting antivirals, protease inhibitor, NS5A inhibitor, sustained viral response, resistance

## Abstract

Sustained virological response (SVR) rates have increased dramatically following the approval of direct acting antiviral (DAA) therapies. While individual DAAs have a low barrier to resistance, most patients can be successfully treated using DAA combination therapy. However, DAAs are vulnerable to drug resistance, and resistance-associated variants (RAVs) may occur naturally prior to DAA therapy or may emerge following drug exposure. While most RAVs are quickly lost in the absence of DAAs, compensatory mutations may reinforce fitness. However, the presence of RAVs does not necessarily preclude successful treatment. Although developments in hepatitis C virus (HCV) therapy in Asia have largely paralleled those in the United States, Japan’s July 2014 approval of asunaprevir plus daclatasvir combination therapy as the first all-oral interferon-free therapy was not repeated in the United States. Instead, two different combination therapies were approved: sofosbuvir/ledipasvir and paritaprevir/ritonavir/ombitasvir/dasabuvir. This divergence in treatment approaches may lead to differences in resistance challenges faced by Japan and the US. However, the recent approval of sofosbuvir plus ledipasvir in Japan and the recent submissions of petitions for approval of paritaprevir/ritonavir plus ombitasvir suggest a trend towards a new consensus on emerging DAA regimens.

## 1. Introduction

### 1.1. Hepatitis C Virus

Hepatitis C virus (HCV) is an enveloped virus in the *hepacivirus* genus of the *Flaviviridae* family. The 9.6 kb positive-sense, single-stranded RNA genome encodes a 3000 nucleotide single polyprotein that is cleaved into three structural proteins and six non-structural proteins. In spite of recent advances in antiviral therapy, hepatitis C virus (HCV) remains a major public health challenge. At least 185 million people throughout the world are chronically infected [1,2], and in Japan the rate of chronic HCV infection is estimated to be up to 2% of the population. Compared to Western countries, Japanese patients are more likely to be older and female and are more likely to be treatment-experienced with more advanced liver disease [3].

### 1.2. HCV Variability

The NS5B RNA-dependent RNA polymerase is highly error prone and leads to frequent substitutions, resulting in high intra-patient variability (1%–5%) represented in the form of quasispecies [4,5]. Inter-patient variability is also high, with six recognized genotypes varying by up to 30%–50% of the sequence and multiple sub-genotypes varying from 15% to 30% [6]. HCV genotype frequencies vary geographically, with genotype 1 being the most common worldwide, followed by genotypes 3, 2, and 4. Genotypes 1 and 4 are considered the most difficult to treat, although the focus of current drug development efforts on genotype 1 has result in high success rates for patients with this genotype. Genotypes 2 and 3 have typically been considered more responsive to treatment and have required shorter durations of interferon therapy, but interferon-free approaches have revealed greater differences between the genotypes, and effective DAA therapy for genotype 3 is currently a major treatment goal.

### 1.3. HCV Treatment

HCV infection is often asymptomatic, but patients with chronic HCV are at greater long-term risk of cirrhosis, liver failure, and hepatocellular carcinoma (HCC). Even patients who have successfully cleared the virus are still at greater risk of developing HCV when liver damage is extensive. However, normalization of serum alfa-protein levels in patients who achieve SVR suggests that effective treatment may reduce the risk of HCC [7]. Therefore, patients with chronic HCV should be identified and treated at an early stage if possible. Until recently, however, the efficacy of the standard of care treatment for genotype 1 remained below 50%, and non-responders had few other treatment options. Treatment success is defined in terms of sustained virological response (SVR) in which the virus remains undetectable 24 weeks after the end of therapy. Interferon-based therapies typically ranged from 24 to 48 weeks depending on the viral genotype, with extension to 72 weeks in some slow-response patients [8]. Some patients showed a transient response to interferon but relapsed during follow-up, whereas other patients showed no change in HCV RNA levels in response to interferon therapy. There are currently four main classes of DAAs: NS3/4A inhibitors, NS5A inhibitors, and both nucleos(t)ide as well as nonnucleoside analogs targeting the NS5B polymerase.

### 1.4. Direct Acting Antiviral Agents

**Figure 1:**
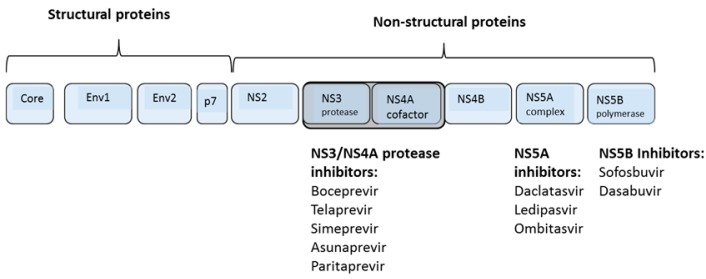
HCV genome architecture and DAA targets. The HCV RNA genome is initially translated as a polyprotein which is initially cleaved by host proteases after which the NS3/NS4A protease cleaves itself and the remaining nonstructural proteins. Direct acting antivirals have been developed against the NS3 protease, the NS5A replication complex, and the NS5B polymerase.

Because HCV does not integrate into the human genome and must replicate continuously to maintain infection, it should be possible to eradicate the virus by blocking replication at one or more stages of the life cycle. This approach to treating HCV was implemented in the form of direct acting antiviral (DAA) therapy, in which high-throughput methods are used to screen drugs that directly target HCV proteins (Table 1 and Figure 1). The introduction of DAA agents has improved SVR rates and shortened treatment duration. DAAs also help to overcome interferon non-responsiveness [9]. DAAs were initially used in addition to peg-interferon plus ribavirin, which improved SVR rates but at the expense of further restricting patient eligibility and increasing the range of side effects. The recent goal of developing interferon-free DAA combination therapies in which two or more DAA classes are co-administered aims to reduce side effects and extend patient eligibility.

**Table 1:** Characteristics and resistance profiles of selected direct acting antiviral agents in the United States and Japan.

**Class**		**Drug**	**Manufacturer**	**Approved for gt 1**
**NS3/4A Protease Inhibitors**				
	**First-Generation, First-Wave**			
		Boceprevir (SCH503034)	Merck	US (2011)
		Telaprevir (VX-950)	Janssen	US (2011); Japan (2011)
	**First-Generation, Second-Wave**			
		Simeprevir (TMC-435)	Tibotec	US (2013); Japan (2013)
		Faldaprevir (BI-201335)	BI	withdrawn (2014)
		Asunaprevir (BMS-650032)	BMS	Japan (2014)
		Paritaprevir (ABT-450/r)	AbbVie	US (2014)
		Danoprevir (ITMN-191, RG 7227)	Roche	
		Sovaprevir (ACH-1625)	Achillion	
		Vedroprevir (GS-9451)	Gilead	
		Vaniprevir (MK-7009)	Merck	
	**Second Generation**			
		Grazoprevir (MK-5172)	Merck	
		ACH-2684	Achillion	
**NS5B Polymerase Inhibitors**				
	**Nucleoside Inhibitors**			
		Sofosbuvir (GS-7977)	Gilead	US (2014); Japan (2015)
		Mericitabine (RG-7218)	Roche	
	**Non-Nucleoside Inhibitors**			
	***Thumb II Inhibitors***			
		GS-9669	Gilead	
		VX-222	Vertex	
		BMS-791325	BMS	
	***Palm I Inhibitors***			
		Dasabuvir (ABT-333)	AbbVie	US (2014)
		ABT-072	AbbVie	
		Setrobuvir (ANA-598)	Roche	
**NS5A Inhibitors**				
	**First Generation**			
		Daclatasvir (BMS-790052)	BMS	Japan (2014)
		Ledipasvir (GS-5885)	Gilead	US (2014); Japan (2015)
		Ombitasvir (ABT-267)	AbbVie	US (2014)
		PPI-668	Presidio	
		PPI-461	Presidio	
		ACH-2928	Achillion	
		GSK-2336805	GlaxoSmithKline	
		BMS-824393	BMS	
		Samatasvir (IDX719)	Idenix	
	**Second Generation**			
		Elbasavir (MK-8742)	Merck	
		ACH-3102	Achillion	
		GS-5816	Gilead	

BMS: Bristol-Myers Squibb; BI: Boehringer Ingelheim.

### 1.5. DAA Resistance

**Figure 2:**
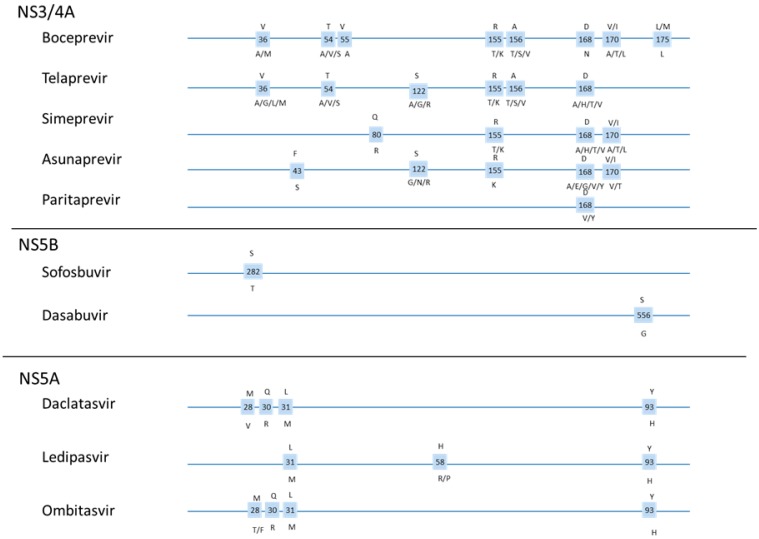
Position of frequently reported resistance mutations for selected DAAs [12].

Although DAA therapies hold great promise as a potential treatment for nearly all patients with chronic HCV, the high specificity of DAAs against their viral targets makes them sensitive to small changes in the sequence of viral peptides, resulting in emergence of antiviral resistance and treatment failure in some patients. Each drug family has a specific resistance profile that influences the barrier to resistance and may vary among genotypes or sub-genotypes (Figure 2). As raw material for selection, the low-fidelity HCV polymerase routinely generates all possible single and double mutations, and the high replication rate of HCV allows minor variants to increase rapidly in frequency in response to drug pressure. Resistance-associated mutants may arise at any time prior to or during therapy. Most variants have lower fitness relative to wild type and are quickly lost, but in the worst case, secondary mutations may compensate for fitness loss and allow the variant to remain even in the absence of the drug. Cross-resistance among DAAs is high, with resistance to one drug often conferring at least partial resistance to other drugs in the same class. Some resistance-associated variants detected in treatment-naive patients appear to be associated with the patient’s IFNL3 genotype, which might indicate selection due to innate immune responses in these patients [10,11].

### 1.6. Resistance Testing

Although methods for detecting resistance-associated variants (RAVs) have been described [13,14] and commercial assays are available for certain variants (e.g., NS3 Q80K), there is as yet no standard way to evaluate patients for the presence of RAVs in clinical practice and few guidelines on their effective use. Direct sequencing is an efficient way to characterize dominant variants but lacks the sensitivity to detect minor variants. While ultra-deep sequencing may be used to characterize novel RAVs, the method is expensive and unsuitable for routine clinical use, and the background error rate of the current methods makes it difficult to discriminate rare variants from sequencing errors. Assay systems designed to detect specific variants (e.g., NS5A Y93) are more practical but are limited due to the high degree of HCV variability. Uchida *et al.* described an assay system to detect NS5A Y93H variants using real-time PCR with primers spanning a 186 nucleotide region of NS5A and four types of oligonucleotides representing the two codons each for tyrosine (Y) and histidine (H) at Y93 [14]. However, this method may fail to detect strains with unanticipated sequence variants at Y93 or in the primer region. Yoshimi *et al.* reported a more robust approach using nested real-time PCR using degenerate probes followed by the Invader reaction [13]. The method was superior to direct sequencing in detecting low-frequency Y93H variants, and Y93H frequencies correlated well with results using deep sequencing. However, the outer primers were ineffective in amplifying cDNA in 9% of patients in spite of relatively high conservation in this region, and long-distance PCR probes using a more conserved region were used instead. Amplification failures in other patients required use of direct sequencing data to in order to select the most appropriate alternative cycling probes. While most patients could be successfully tested after these manual steps, the high variability of the HCV genome makes automation and routine use of such tests more difficult. However, assay systems should become more accurate and sensitive as additional sequence variability data becomes available. Unlike current Japanese guidelines that recommend pre-treatment RAV testing, current EASL and AASLD treatment guidelines do not recommend pre-treatment resistance testing in treatment-naive patients except in the case of NS3 Q80K variants in simeprevir-based therapy [15,16]. However, future guidelines may expand recommendations on the role of pre-treatment resistance testing and consideration of prior DAA treatment history in guiding treatment decisions.

## 2. NS3/4A Protease Inhibitors

### 2.1. NS3/4A Protease

After translation of the viral genome, the 3000 amino acid polyprotein must be cleaved into three structural proteins and six non-structural proteins. The structural proteins are cleaved by cellular proteases, but the non-structural region encodes a protease, which cleaves first itself and then the remaining non-structural proteins. The NS4A protease and its cofactor NS3 form a heterodimer localized to the endoplasmic reticulum. Aside from its essential role in viral infection, the protease also plays a role in immune suppression by cleaving two key interferon signaling molecules [17,18,19].

### 2.2. Telaprevir and Boceprevir

In July 2011, the FDA approved two NS3/4 serine protease inhibitors (PIs), telaprevir and boceprevir. These α-ketoamide electrophilic trap-containing inhibitors mimic the carboxy-terminal region of the HCV NS3/4 serine protease and reversibly target S139 of the active site [20,21]. Telaprevir monotherapy is impractical due to rapid emergence of resistance variants [22,23], but telaprevir greatly increased SVR rates when used in combination with peg-interferon plus ribavirin. However, this demanding therapy further restricted patient eligibility, and patients undergoing the therapy often experienced adverse events.

### 2.3. Telaprevir Resistance

The NS3 domain is highly divergent among HCV genotypes, but first generation PIs are highly specific to genotype 1, which not only limits their use against other HCV genotypes but also results in a relatively low barrier to resistance [24]. Even within genotype 1, resistance occurs more frequently in genotype 1a than 1b due to a synonymous codon at R155 that reduces the number of nucleotide changes required to cause an amino acid substitution [25]. While these mutations tend to have lower fitness than wild type and cannot compete in the absence of the drug, compensatory mutations such as V36M restore viral fitness, allowing the virus to compete in the absence of the drug and increasing the risk of persistence [26]. Discontinuation of telaprevir or boceprevir therapy is recommended in the event of viral breakthrough, but resistant strains are also highly cross-resistant to other PIs in the same class, potentially confounding future treatment efforts. The shallow orientation of the NS3 catalytic site prevents tight binding of inhibitors and PIs rely on interaction with several key residues [27]. Although resistance profiles differ slightly among current PIs, most first generation PIs are vulnerable to R155 and D168 substitutions. First generation PIs are ineffective against genotype 3 because most strains are fixed for the D168Q substitution.

### 2.4. Telaprevir Triple Therapy in Japan

Telaprevir, although not boceprevir, was approved in Japan in September 2011. Telaprevir triple therapy entered widespread clinical use and resulted in improved rates of SVR. However, the therapy was poorly tolerated, and many patients experienced adverse effects including rash and anemia. Ribavirin dosage is determined by body weight, and in the case of anemia, the dosage may be reduced without compromising safety, whereas telaprevir dosage is fixed. While initial dose-determining studies were performed mainly in Europe and North America, Japanese patients tend to have lower average body weight, which may have contributed to the high incidence of adverse events.

### 2.5. Second Wave Protease Inhibitors

While the second generation of PIs to overcome these obstacles is under development, incremental improvements to increase the barrier to resistance and extend coverage to other genotypes has led to a second wave of improved first generation PIs. Major goals in the design of these drugs included improved safety and tolerability profiles and improved patient compliance through a reduced pill burden [28]. In a sign of the rapid pace of DAA development, Vertex Pharmaceuticals discontinued sales of telaprevir in October 2014, and Merck has announced that it will discontinue sales of boceprevir by December 2015, due mainly to reduced demand for the drugs following the approval of alternative DAAs.

### 2.6. Simeprevir

In November 2013, the FDA approved simeprevir (TMC-435), a macrocyclic PI with a once per day dosing regimen and antiviral activity against genotypes 1, 2, 4, 5, and 6 [29]. A 150 mg dose was approved in the US, but a lower 100 mg dose was approved in Japan. Like telaprevir, simeprevir was approved for use in combination with peg-interferon and ribavirin, but SVR rates were higher and incidence of adverse events was lower than in telaprevir triple therapy, although rash and photosensitivity are more common than with peg-interferon and ribavirin alone. Simeprevir also has the advantage of once-daily dosing, compared to telaprevir’s twice or three times daily dosing, and unlike telaprevir, it is not required to be ingested with a high-fat meal. Inhibition of the bilirubin transporter organic anion transporter protein B1 (OATP1B1) leads to elevation of serum bilirubin in some patients, but simeprevir is 1000 times less active against 20 human proteases than against the viral protease [21,30]. Simeprevir can also be given to liver transplant recipients without dose adjustments for cyclosporine or tacrolimus. In spite of these improvements, simeprevir is vulnerable to antiviral resistance (Q80, S122, R155, and D168) and should not be used in patients who failed to respond to prior PI therapy due to cross-resistance. SVR was also significantly reduced in genotype 1a patients in whom Q80K variants were present at baseline.

### 2.7. Asunaprevir (BMS-650032)

In July 2014, Japan approved asunaprevir for use in combination with the NS5A inhibitor daclatasvir, making it the first approved all-oral, interferon/ribavirin-free DAA therapy [31]. Another of the first generation second-wave PIs, asunaprevir has improved safety with once or twice daily oral administration and antiviral activity against genotypes 1, 4, 5, and 6. Although the barrier to resistance is higher than in first wave PIs, asunaprevir resistance has been reported for several variants: F43S, S122G/N/R, R155K, D168A/E/G/V/Y, and V170T. Although asunaprevir is being used successfully in Japan, due to differences in genotype frequencies between the two populations and faced with increasing competition from Gilead (Foster City, CA, US) and AbbVie (North Chicago, IL, US), Bristol-Myers Squibb (New York, NY, US) has withdrawn its application for asunaprevir in the US.

### 2.8. ABT-450/r (Paritaprevir with Ritonovir)

While asunaprevir will not appear in the US market, another second wave PI, ABT-450, was approved by the FDA in December 2014. Because ABT-450 is a substrate of cytochrome P450, it is co-administered with the cytochrome P450 inhibitor ritonavir, which increases the half-life and permits once daily dosing [32]. Ritonavir interacts with the HIV protease, complicating the drug’s use in HIV-positive patients and requiring full suppression of HIV RNA prior to therapy. High doses of the drug help to suppress development of resistance, but the risk of resistance is higher in genotype 1a compared to 1b. In a phase III clinical trial of 473 patients, NS3 D168V variants were detected in seven out of the eight patients who experienced virologic failure or relapse [33].

### 2.9. Second Generation PIs

Although second wave drugs incorporate numerous improvements over the first wave PIs, they remain cross-resistant and share a low barrier to resistance. The term second generation is reserved for PIs that are unaffected by resistance variants affecting first generation PIs, and have pan-genotypic activity against a range of HCV genotypes, including genotype 3. While several drugs, such as MK-5172 and ACH-2684, are undergoing clinical trials, no second generation PIs have yet been approved in the US or Japan.

## 3. NS5B Polymerase Inhibitors

### 3.1. NS5B RNA-Dependent RNA Polymerase

While PIs act at an early stage of the HCV life cycle by interfering with cleavage of the polyprotein, other DAAs target different stages of the life cycle. Polymerase inhibitors target the HCV NS5B RNA-dependent RNA polymerase (RdRp/NS5B). This low-fidelity polymerase synthesizes a negative strand RNA, which is then used to produce multiple positive strand copies of the HCV genome for replication and translation [34]. Two different types of polymerase inhibitors, nucleoside inhibitors (NIs) and non-nucleoside inhibitors (NNIs) have been developed. A number of NI and NNI candidates have reached advanced clinical trials in humans but were later withdrawn, including the NIs IDX-184, INX-189, and GS-6620 and the NNIs deleobuvir, tegobuvir, and filibuvir [16].

### 3.2. Nucleoside Inhibitors

NIs are similar to naturally occurring nucleotides, but they inhibit the RdRp active site and cause chain termination when they are incorporated into the elongating RNA sequence [35,36]. NIs are administered as a prodrug that must be phosphorylated to become an active nucleoside triphosphate. In principle, NIs have a low barrier to resistance because single amino substitutions are able to confer resistance, but in practice, the barrier to resistance is relatively high because the active site is strongly conserved among all HCV genotypes and resistance variants tend to have poor fitness [35]. In fact, NIs have the highest barrier to resistance among the DAAs available so far [28]. However, NS5B resistance mutations have been observed in DAA-naive patients. In a direct sequencing study of DAA treatment-naive HCV patients, V321I, M426L, Y448H, Y452H were detected in 13% of genotype 1a patients and L159F, V321I, C316N, M426L, Y452H, R465G and V499A mutations were detected in 90% of genotype 1b patients [37]. While many of these mutations confer only a low level of resistance, they highlight the extreme variability of the HCV genome and suggest a deep bench of potential DAA resistance variants.

### 3.3. Sofosbuvir (GS-7977)

One of the most important new DAAs, the NI polymerase inhibitor sofosbuvir was approved in the US in December 2013 under its breakthrough therapy designation based on results from multiple clinical trials, including FISSION, NEUTRINO, POSITRON, and FUSION [38,39,40]. The sofosbuvir prodrug is converted into GS-331007, which is then taken up by hepatocytes and converted by cellular kinases to the active form, GS-461203 [41]. The FDA approved a 12 week course of once daily 400 mg sofosbuvir in combination with peg-interferon and ribavirin for treatment of genotypes 1 and 4 or with ribavirin alone for the treatment of genotypes 2 (12 weeks) and 3 (24 weeks). SVR rates greater than 90% have been reported with adverse events such as fatigue and headache on par with peg-interferon and ribavirin alone [39]. Although HCV genotyping has long been performed to guide therapy decisions, discordances in genotyping methods in sofosbuvir clinical trials have revealed a higher than expected incidence of HCV recombinant strains, particularly RF1_2k/1b, in which the response to therapy was more similar to genotype 1 than genotype 2 due to the presence of genotype 1 NS5B [42,43]. Given the genotype-specific differences in the content and duration of therapy, HCV genotyping might require greater scrutiny prior to DAA therapy. Although co-administration of the drug with amiodarone and another DAA, such as ledipasvir or simeprevir, has led to rapid-onset brachycardia in a small number of patients, sofosbuvir is well tolerated and has been successfully used to treat patients with HCV/HIV co-infection. No dose reduction is necessary for elderly patients, but Gilead suggests that 24 weeks of therapy with sofosbuvir and ribavirin could be used to treat interferon-ineligible patients with genotype 1, and up to 48 weeks of sofosbuvir plus ribavirin therapy could be used to reduce the risk of post-transplant re-infection in patients awaiting liver transplantation. Ribavirin dose modifications in response to hemoglobin abnormalities are common, but dose reduction of sofosbuvir is not recommended, and sofosbuvir should be discontinued in the event that other ribavirin and other agents are discontinued. Sofosbuvir therapy is also not recommended in patients with severe renal impairment or end stage renal disease due to twenty-fold greater exposure of the metabolite. Conversely, intestinal exposure to P-gp inducer drugs such as rifampin and St. John’s wort decreases the plasma concentration of sofosbuvir and may compromise the effectiveness of therapy. In November 2014 the FDA approved sofosbuvir plus simeprevir as an interferon-free dual DAA therapy for genotype 1, and in July 2015, sofosbuvir was approved in Japan in combination with ledipasvir for treatment of genotype 1 and in combination with ribavirin for treatment of genotype 2.

Although cost has long been a concern in HCV therapy, sofosbuvir pricing in particular has attracted controversy. The US $84,000 cost of a 12 week course of treatment, averaging $1,000 per 400 mg pill, has raised concern about the drug’s cost effectiveness. The World Health Organization recently added sofosbuvir to its list of essential medicines and urged lower prices. While the drug is available at lower cost in Europe and is offered in generic form or at steeply discounted rates in 91 developing countries, as low as 1% of the US price, the drug nonetheless remains out of reach for 59 million people around the world. While Gilead holds a patent on sofosbuvir in China, India rejected Gilead’s patent claim for Sovaldi, and China recently rejected Gilead’s patent application for the prodrug, opening the door to further patient challenges and generics. Medical tourism may be an unintended consequence of this pricing differential, as demand for the drug has encouraged patients to seek more affordable treatment overseas. After failing to negotiate a sufficient discount, the largest US pharmacy benefit manager discontinued coverage of the drug, opting for AbbVie’s paritaprevir/ritonavir/ombitasvir/dasabuvir Viekira Pak instead. However, the cost to develop a new DAA is high, and in the pace of rapid development, the effective life span of a new DAA may be limited. Therefore, at this stage in DAA development, restricting the availability of effective treatment options based on pricing considerations alone may be shortsighted. Given the drug’s efficacy and the low occurrence of resistance, sofosbuvir is poised to play a key role in managing resistance in the near future, especially when additional combination therapies with other DAAs are approved.

### 3.4. Non-Nucleoside Inhibitors

While NIs act by directly interfering with the RdRp active site, NNIs suppress RdRp activity indirectly by binding to one of several known allosteric sites located away from the active site [44]. NNIs are currently only effective against genotype 1 and have lower antiviral potency and a lower barrier to resistance than NIs, especially in genotype 1a in which the drug dissociates faster than in 1b [45]. Resistance mutations also do not necessarily compromise viral fitness, making it easier for the variant to persist in competition with the wild-type strain. However, NS5B contains at least five druggable targets, and several NNIs under investigation, including the thumb I/II and palm I/II inhibitors, may provide complementary protection by targeting different regions of the molecule [45].

### 3.5. ABT-333 (Dasabuvir)

The NNI dasabuvir was approved by the FDA in December 2014 for use in combination with paritaprevir/ritonavir (PI) and ombitasvir (NS5A inhibitor) with or without ribavirin for treatment of adults with genotype 1, including patients with compensated cirrhosis. The twice daily dosing regimen is higher than some other combinations but is simplified using the Viekira Pak, which contains one tablet of dasabuvir and one tablet co-formulated with ombitasvir, paritaprevir, and ritonavir. Ribavirin is not required but is recommended for all patients except non-cirrhotic patients with genotype 1b. SVR rates between 91% and 100% were achieved in clinical trials involving 2308 patients [46]. The cost of a 12-week Viekira Pak is $83,319. Except for ritonavir, which had been previously approved as an HIV protease inhibitor, none of the drugs in the Viekira Pak have been approved for standalone use. In a phase III trial involving 473 patients, NS5B-S556G resistance variants were detected in four patients who failed to achieve SVR [33].

## 4. NS5A Inhibitors

### 4.1. NS5A

NS5A inhibitors form a cornerstone of most DAA combination therapies. Non-structural protein 5 (NS5A) is a 447 amino acid viral phosphoprotein. Even though it has no known enzymatic activity, making it an unusual drug target, NS5A plays a critical role in viral replication [47]. The NS5A dimer forms an essential component of the replication complex but is also thought to contribute to immune evasion and interferon resistance by interfering with signal transduction. Therefore, targeting the multifunctional NS5A should not only disrupt viral replication but may also strengthen the cell’s innate immune response against the virus.

### 4.2. Daclatasvir

To identify potential drug candidates using an unbiased chemical genetics approach, Bristol-Myers Squibb used a high-throughput replicon system to screen over a million small molecules for antiviral activity against HCV [47,48]. Candidate compound BMS-858 was found to weakly inhibit replication of HCV Con-1 genotype 1b in Huh-7 liver cells. The compound was extensively refined to improve potency, availability, and genotypic coverage, resulting in the development of daclatasvir (BMS-790052). Daclatasvir showed potent antiviral activity and reduced serum HCV RNA by 3.3 log_10_ IU/mL within 24 h of administration of a single dose. EC_50_ was only 0.009 nM for genotype 1b but somewhat higher at 0.050 nM for genotype 1a [49]. While efficacy of most first generation drugs is limited to genotype 1, daclatasvir showed potent antiviral effects against genotypes 1–6 *in vitro* [47,48]. Although the drug is relatively large (>700 g/mol), it is delivered efficiently to the liver and is well tolerated at doses up to 100 mg, permitting once daily dosing [49,50,51]. Daclatasvir is thought to target a region in the first 100 amino acids of NS5A, perhaps disrupting protein function by interfering with dimer association [47].

Daclatasvir was developed as part of an interferon/ribavirin-free dual therapy in combination with asunaprevir (PI) [52,53]. In an open-label phase III clinical trial in Japan, 222 genotype 1 patients were treated with asunaprevir plus daclatasvir for 24 weeks [54]. All patients were either prior null responders or were ineligible for interferon therapy. SVR rates ranged from 81% in prior non-responders to 91% in cirrhotic patients. The therapy was approved in July 2014 in Japan. Given its effectiveness and low incidence of adverse events, the therapy has become widely used in Japan, and about 40,000 patients have been treated so far. However, about 10% of those treated have developed resistance. Daclatasvir resistance mutations corresponded to those previously characterized *in vitro*, including L31 and Y93 (genotype 1a and 1b) and M28 and Q30 (genotype 1a). Both daclatasvir and asunaprevir-resistant substitutions were detected by direct sequencing in patients with treatment failure [55]. While asunaprevir-resistant variants were no longer detectable 48 weeks after the end of treatment, daclatasvir-resistant variants remained. Although NS5A-resistant mutations can emerge after exposure to the drug, they are also often naturally present, at least at low frequency, in DAA-naive patients [37]. Paolucci *et al.* observed M28V, L31M, and H58P in 13% of DAA-naive genotype 1a patients and L28V, L31M, Q54H, Y93H, and I280V in genotype 1b patients [37]. The frequency of NS5A inhibitor-resistant variants in DAA-naive patients is about 4% worldwide but about 11%–23% in Japan’s genotype 1b patient population [56]. In a study examining the emergence of daclatasvir RAVs using ultra-deep sequencing, 31 patients were treated with daclatasvir plus asunaprevir for 24 weeks [7]. Two patients experienced viral breakthrough and two patients relapsed. While no NS3-D168 RAVs were detected prior to treatment, NS5A L31M or Y93F/H RAVs were detected in 30% of the patients prior to therapy, of whom only 5 out of the 9 achieved SVR. NS3-168 RAVs were rapidly lost after discontinuation of therapy, but NS5A variants remained at high frequency. Patients with pre-existing NS5A mutations or who experienced on-treatment emergence of NS3 and/or NS5A variants were more likely to experience treatment failure. Persistence of NS5A variants may complicate retreatment efforts in these patients, although *in vitro* studies suggest a number of all-oral treatment options for patients who fail to achieve SVR under daclatasvir plus asunaprevir therapy [57]. The presence of NS5A variants does not necessarily ensure treatment failure, although the high pre-treatment frequency of NS5A RAVs in Japanese patients suggests that patients should be screened prior to NS5A treatment.

### 4.3. Ledipasvir (GS-5885)

Several other NS5A inhibitors have undergone clinical trials in Japan. A recent multi-centre, open-label, randomized phase III clinical trial (NCT01975675) evaluated the effect of 12 weeks of Gilead’s NS5A inhibitor ledipasvir and sofosbuvir (NS5B inhibitor) with or without ribavirin in 341 Japanese patients with chronic genotype 1 infection [58]. All patients who received ledipasvir and sofosbuvir achieved SVR12, and 98% of patients who received ledipasvir, sofosbuvir, and ribavirin achieved SVR12. NS5A Y93H resistance variants were detected in 76 patients prior to therapy, although all but one achieved SVR12. In July 2015, Japan’s Ministry of Health, Labour, and Welfare approved Harvoni, which combines sofosbuvir and ledipasvir in a once-daily single tablet, for treatment of patients with genotype 1.

### 4.4. Ombitasvir (ABT-267)

Co-formulated as a single tablet with the PI paritaprevir/retonavir and administered along with ribavirin and the NNI dasabuvir, ombitasvir is an NS5A inhibitor with picomolar, pan-genotypic efficacy. In a phase III study of 473 patients treat with this combination therapy, one patient had virologic failure and seven patients relapsed [33]. In each case, RAVs against at least one of DAA were detected, including NS5A-M28T (two patients) and Q30R (three patients) in patients with genotype 1a and L31M and Y93H in the patient with genotype 1b.

### 4.5. Ombitasvir and Paritaprevir/Ritonavir

A recent open-label, phase II, randomized clinical trial was conducted in Japan to evaluate dual therapy with 12 or 24 weeks of ombitasvir (NS5A inhibitor) and paritaprevir/ritonavir (PI) in 101 patients with genotype 1b or 2 who failed to respond to prior peg-interferon plus ribavirin combination therapy [59]. Because of the high frequency of genotype 1b in Japan, ribavirin was not included. SVR rates ranged from 89% to 100% in genotype 1 patients depending on dosage and treatment duration, and slightly lower SVR rates at 58% and 72% in genotype 2 patients. However, SVR rates were 90% in genotype 2a patients compared to 27% in genotype 2b patients. The one genotype 1b patient who relapsed had NS3 D168V and NS5A Y93H RAVs at the time of failure but not prior to treatment. Although NS5A Y93H was present at baseline in four genotype 1b patients, all nonetheless achieved SVR. RAVs including NS3 D168V/Y and NS5A L28F were detected in all genotype 2 patients who experienced virologic failure, but the presence of baseline RAVs did not influence treatment outcome. Ombitasvir and paritaprevir/ritonavir dual therapy was submitted for marketing approval in Japan in February 2015 and upgraded to priority review in April 2015.

## 5. Conclusions

The large number of DAAs approved or pending approval signals a clear trend away from interferon as a first line therapy in the treatment of HCV. Interferon lambda might have played a larger role in treatment of HCV were it not for the timely introduction of DAAs, but interferons may continue to play a useful role in treating or conditioning patients with DAA resistance, as interferon’s broad antiviral activity may help to clear resistant strains, improving the chance of successful re-treatment with DAA therapy. Treatment guidelines for patients with emergent RAVs have not been fully established, although DAA therapy should be discontinued in such patients. The most recent Japanese guidelines recommend pre-treatment analysis of NS5A L31/Y93 RAVs prior to daclatasvir plus asunaprevir therapy, while current EASL and AASLD treatment guidelines do not recommend pre-treatment resistance testing except in the case of NS3 Q80K variants in simeprevir-based therapy. However, AASLD guidelines suggest that treatment resistance testing should be performed prior to re-treatment in patients for whom prior NS5A therapy had failed, although no specific recommendations are provided [14,15]. Perhaps such patients could be treated with peg-interferon plus ribavirin therapy as a lead-in therapy to restore wild type frequencies prior to attempting further DAA therapy. New DAA therapies will likely include at least two of the four current classes of DAAs. Except for the Viekira Pak, current therapies typically include an NS5A inhibitor and either an NS5B inhibitor (USA) or a PI (Japan), but future therapies seem likely to include sofosbuvir. The continuing role of ribavirin in the DAA era is not clear, and results from a number of clinical trials have failed to show that addition of ribavirin significantly improves SVR rates. The FDA continues to require ribavirin in several therapies, whereas ribavirin use in Japan appears to be decreasing due its toxicity and the efficacy of ribavirin-free therapies in Japan’s predominantly genotype 1b patient population. The large number of approved and pending DAAs in each country makes for a confusing treatment landscape, but several drugs, including sofosbuvir, ledipasvir, ombitasvir, and paritaprevir, appear poised to play leading roles in emerging HCV treatments in both countries, as well as in Europe. However, antiviral resistance, costs, and competition from other drug makers are likely to maintain the current dynamic state of HCV therapy for several years to come.
